# Nanofabrication of Conductive Metallic Structures on Elastomeric Materials

**DOI:** 10.1038/s41598-018-24901-2

**Published:** 2018-04-26

**Authors:** Edward K. W. Tan, Girish Rughoobur, Juan Rubio-Lara, Nikhil Tiwale, Zhuocong Xiao, Colin A. B. Davidson, Christopher R. Lowe, Luigi G. Occhipinti

**Affiliations:** 1 Department of Engineering, University of Cambridge, Cambridge, CB3 0FA UK; 2 Microsystems Technology Laboratories, Massachusetts Institute of Technology, Cambridge, MA 02139 USA; 3 Nanoscience Centre, University of Cambridge, Cambridge, CB3 0FF UK; 4 Institute of Biotechnology, University of Cambridge, Cambridge, CB2 1QT UK

## Abstract

Existing techniques for patterning metallic structures on elastomers are limited in terms of resolution, yield and scalability. The primary constraint is the incompatibility of their physical properties with conventional cleanroom techniques. We demonstrate a reliable fabrication strategy to transfer high resolution metallic structures of <500 nm in dimension on elastomers. The proposed method consists of producing a metallic pattern using conventional lithographic techniques on silicon coated with a thin sacrificial aluminium layer. Subsequent wet etching of the sacrificial layer releases the elastomer with the embedded metallic pattern. Using this method, a nano-resistor with minimum feature size of 400 nm is fabricated on polydimethylsiloxane (PDMS) and applied in gas sensing. Adsorption of solvents in the PDMS causes swelling and increases the device resistance, which therefore enables the detection of volatile organic compounds (VOCs). Sensitivity to chloroform and toluene vapor with a rapid response (~30 s) and recovery (~200 s) is demonstrated using this PDMS nano-resistor at room temperature.

## Introduction

Electronics made on elastomers represent an emerging class of technology that have found applications in various areas including electronic skin^1^, flexible solar cell^2^, stretchable display^3^, bendable touchpad^4^, implantable neuroelectrode^5^, neural prosthesis^6^, electronic eye camera^7^, and wearable sensor for continuous monitoring^8–10^. Polydimethylsiloxane (PDMS) plays a crucial role in such devices, serving as an encapsulation^11^, dielectric^12^ or substrate^13^ due to its desirable properties such as low electrical conductivity, easy preparation, biocompatibility, deformability and chemical inertness in the body environment^14^. However, one undesirable characteristic of PDMS is its affinity towards organic compounds as the silicone could swell massively upon contact with the liquids^15,16^. As a result, complications arise during device fabrication, as organic solvents/vapors are often used.

Nevertheless, the swelling properties of PDMS could prove useful as an active layer for gas sensing applications. Martinez Hurtado *et al*. demonstrated that a holographic sensor for volatile organic compounds (VOC) could be made with PDMS by laser ablation of the diffused silver salts in the polymeric network^17^. The response was recorded as a shift in wavelength in the elastomer as the position of the fringes altered. Similarly, Park *et al*. showed an optical sensor based on PDMS coated fiber Bragg grating^18^. However, these optical sensing technologies would require a separate light source to work. More recently, Rumens *et al*. reported a passive radio frequency identification device (RFID) which utilized PDMS as a mechanical actuator but this technique has a slow response time and low sensitivity^19^. Gravimetric sensing techniques using bulk (either solidly mounted resonators or quartz crystal microbalances) or surface acoustic waves with PDMS as the sorbent material for VOCs to produce small mass changes that are ultimately detected as frequency shifts have also been demonstrated^20–22^. However, using an additional coating layer on mass-sensitive devices causes acoustic wave leakage, which damps the resonance and hence reduce the device sensitivities^22^. In this article, we introduce a simple concept for the detection of VOC by the direct fabrication of a thin film nano-resistor onto PDMS, which acts as both the sorbent and the substrate material simultaneously.

Direct fabrication of thin metallic films with high resolution features on PDMS is challenging^23^. The large coefficient of thermal expansion (CTE) (~0.31% per K) of PDMS compared to conventional photoresists causes significant expansion mismatch that results in the crack formation during lithography^24–27^. The alternate solution of using wet etching of the metal deposited on PDMS leads to undercutting due to the isotropic etching reaction, which restricts both the resolution and density of the electrodes^28–30^. Metal deposition via conventional shadow masks has also been reported but is limited to large features (>100 µm) and simple designs^31,32^. More recently, Zhang *et al*. have proposed the use of ultrathin parylene films (2 μm thick) as shadow masks to pattern microelectrodes but still achieved only 5 μm in channel length (spacing between electrodes)^33^. Carrier substrates have also been used to transfer patterns from silicon (Si) to elastomeric substrates but such thin polymer films are generally difficult to handle^10,34^. Similarly, pattern transfer through dry peeling damages devices in the nanoscale due to the stress induced by the process to the devices^35^. Therefore a technique to achieve low-stress and high-resolution electrodes on elastomers is a significant advancement.

In this work we demonstrate a fabrication strategy that overcomes the current limitations of reported techniques. We employ a thin aluminium (Al) sacrificial layer as a temporary substrate that is compatible with conventional lithographic techniques. The key properties of this Al layer are: (*i*) the ease of removal with minimal stress, and (*ii*) template stripping can be applied to create a low roughness surface for high resolution patterning. Hence, a conducting device in the nanometer dimension range can be transferred onto a range of spin casted polymers such as PDMS. Based on this fabrication process, we demonstrate a standalone sensing mechanism for VOCs based on direct fabrication of high resolution nano-resistor on PDMS. This proof of concept study explores a miniaturized sensor, with resolution in the nanometer range, which responds to the swelling of PDMS. We also propose a “mesh” design for a wide coverage both in the vertical and in the horizontal directions, and hence higher sensitivity in response to the swelling in the elastomeric film. To validate the proof-of-concept device for VOC detection, we tested the nano-sensor device against VOCs with health implications such as chloroform and toluene.

## Results and Discussions

**Figure 1:**
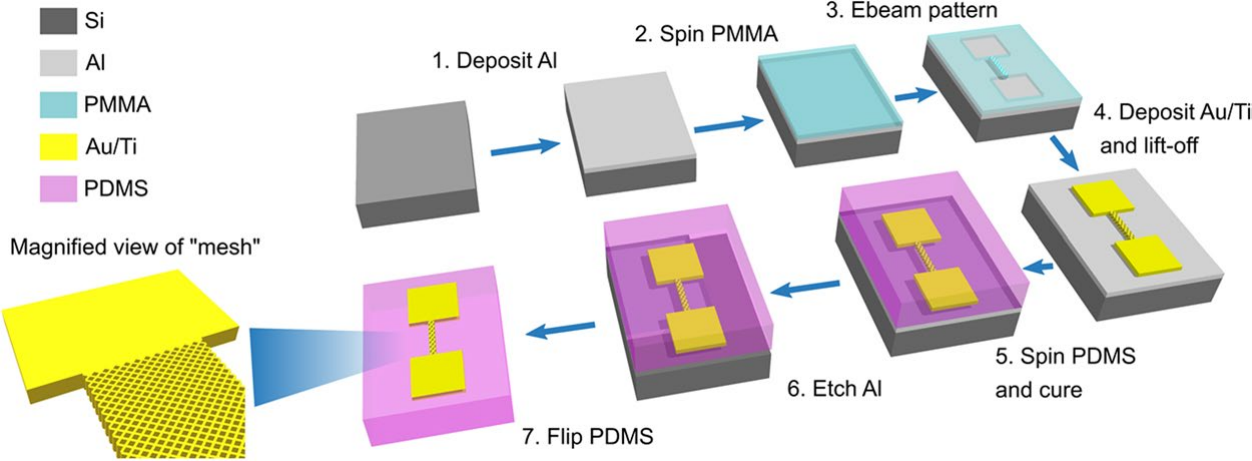
Transfer of high resolution gold patterns down to the nanometer range onto the elastomer via Al sacrificial layer, the steps involve (1) the sputtering of Al on Si, (2) spin-coating PMMA, (3) patterning PMMA with electron beam lithography, (4) deposition of Au/Ti and lift-off, (5) spin-coating and curing PDMS, before (6) etching the Al sacrificial layer to (7) transfer the pattern onto the PDMS.

Figure 1 illustrates the key steps to fabricate high resolution gold (Au) patterned conductive structures onto the PDMS. We first sputtered ~150 nm of Al onto a blank Si substrate as a rigid sacrificial layer. We subsequently define the patterns used for gas detection by electron beam lithography (EBL) and deposited 50 nm of Au and 4 nm of titanium (Ti) via e-beam evaporation. Ti is necessary for adhesion of the Au on the Al surface and can be removed after the transfer. After lift-off, we immersed the samples in 3-mercaptopropyl trimethoxysilane (MPTMS) solution to create a stable bond between Au and PDMS as reported in previous studies^36,37^. We then spin-casted degassed PDMS on the patterned Al and cured the samples at room temperature. Finally, we etched the sacrificial layer in dilute hydrochloric (HCl) solution to release the Au/Ti embedded PDMS with minimal induced stress.

**Figure 2:**
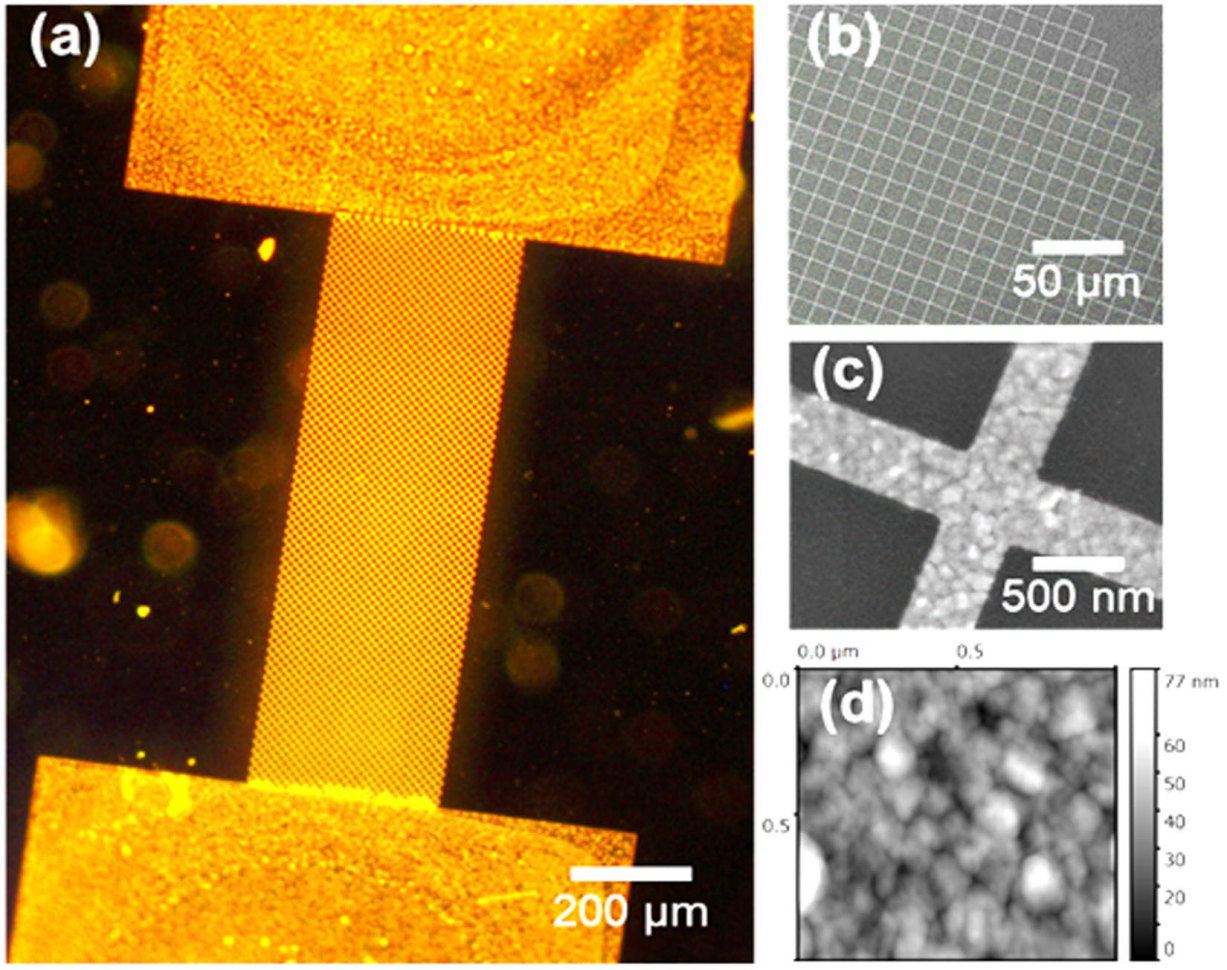
(**a**) Dark field optical image of the resistor “mesh design” (**b**) Scanning Electron Microscope (SEM) image of the mesh network of the device (**c**) Higher magnification of SEM of the mesh (**d**) Atomic force microscope (AFM) image of the roughness inherited due to the sacrificial Al layer.

Figure 2(a) and (b) show the dark field optical image and scanning electron microscope (SEM) image of the Au mesh patterned on PDMS respectively. These structures remained highly conductive on PDMS, suggesting that there was minimal or no fracture of the metallic layer^38^. As compared to previous studies, wrinkling or buckling of thin metal films on PDMS were not observed (see Supporting Information Figure S1) in our method, as the elastomer was not subjected to heat treatment^39–41^. This is an important feature for the resistive based sensor due to the limited stretchability. Furthermore, heating of PDMS would exert excessive thermal stress on the patterned nanostructures, which would lead to the loss of connection within the mesh. Figure 2(c) shows a magnified view of the device, and the width of the mesh was measured to be ~400 nm. Granular structures (average grain size of 111 nm in diameter) of the Au films were observed as the metal was grown directly on the sputtered Al. The physical vapor deposition of metals such as Al is governed by the nucleation, island formation rates, and energy density of the metallic atoms arriving on the substrate^42^. As a result, Al sputtered onto a flat surface such as Si would have a significant root mean square (rms) surface roughness (~9.6 nm) as observed in Fig. 2(d), and this rms roughness can be increased with the deposition temperature^43^. Depositing this Al sacrificial layer at higher temperatures could also increase the surface roughness that the Au layer inherits, and would potentially improve the stretchability of devices fabricated on elastomers. However, this will be achieved at the expense of a loss in resolution when fabricating dense nanoscale features on uneven surfaces, as this is limited by electron beam/UV light scattering.

**Figure 3:**
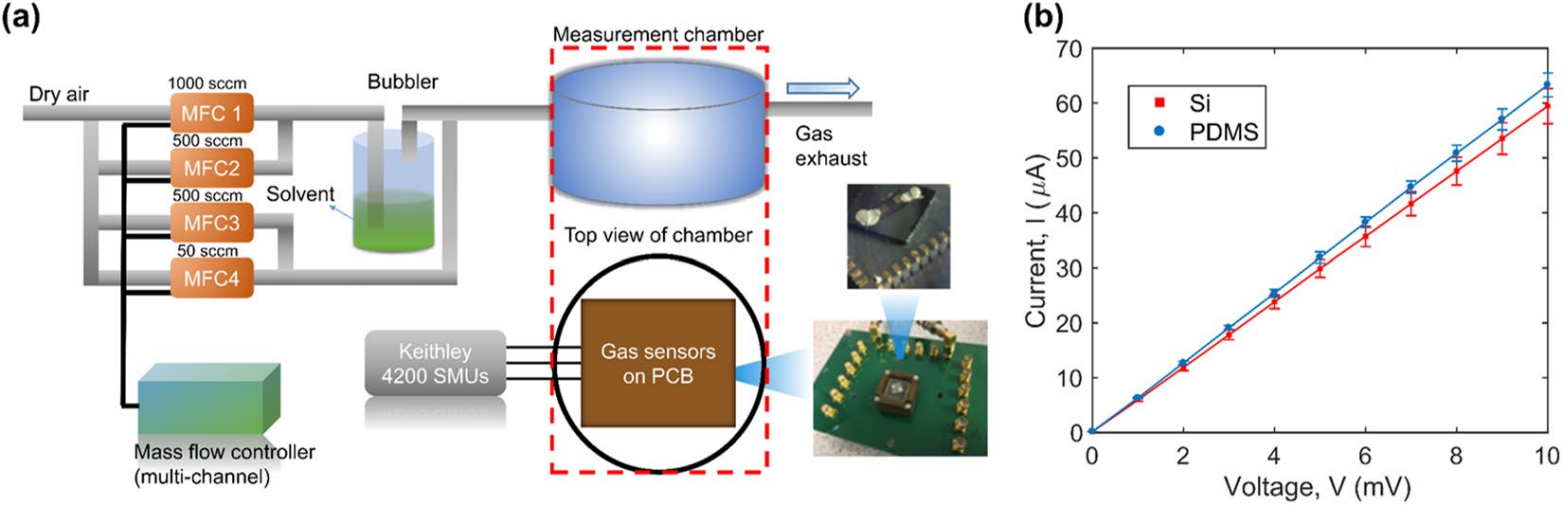
(**a**) Schematic of gas sensing setup demonstrating the connection of the device to a printed circuit board PCB. (**b**) Shows the I-V characteristics of the fabricated nano-resistor on PDMS and Si in ambient conditions.

We then characterized the electrical response (current, I versus voltage, V) of the device under controlled environment using the setup shown schematically in Fig. 3(a). For the tests we connected our device onto a printed circuit board (PCB) via both silver paste and thin copper leads. We then loaded the device in a custom-made gas test chamber, which was sealed with O-rings while the PCB was connected to Keithley Source Measurement Units (SMUs) located outside the measurement chamber as shown schematically in Fig. 3(a). Using this set-up, we compared the I-V characteristics of the mesh structure transferred on PDMS to that of similar devices fabricated directly on Si. We observed consistent electrical responses between the devices made on PDMS and their Si counterparts as shown in Fig. 3(b) where values of electrical resistance, *R* (V/I), of 146 Ω and 150 Ω were measured on PDMS and on Si respectively. Using calibrated mass flow controllers (MFCs) we performed gas sensing measurements of chloroform, toluene, isopropyl alcohol (IPA) and water vapor at various concentrations, achieved via dry air dilution.

In our experiments, we fixed a continuous total flowrate of 500 sccm into the system. We defined 500 sccm flow of dry air as a baseline for the sensor. We directed the entire airflow to the bubbler, as shown in Fig. 3(a), to displace the saturated vapor of chloroform, toluene, IPA and water with respective concentrations of 4.40 × 10^5^ ppm, 3.80 × 10^4^ ppm, 6.36 × 10^4^ ppm and 2.43 × 10^4^ ppm for 30 seconds. The device was then allowed to recover in the flow of dry air for 10 minutes before the next pulse. We measured consistent electrical responses across the measurements of the same compound but different behavior towards different vapors, due to the distinct interactions of these molecules with the PDMS active layer.

**Figure 4:**
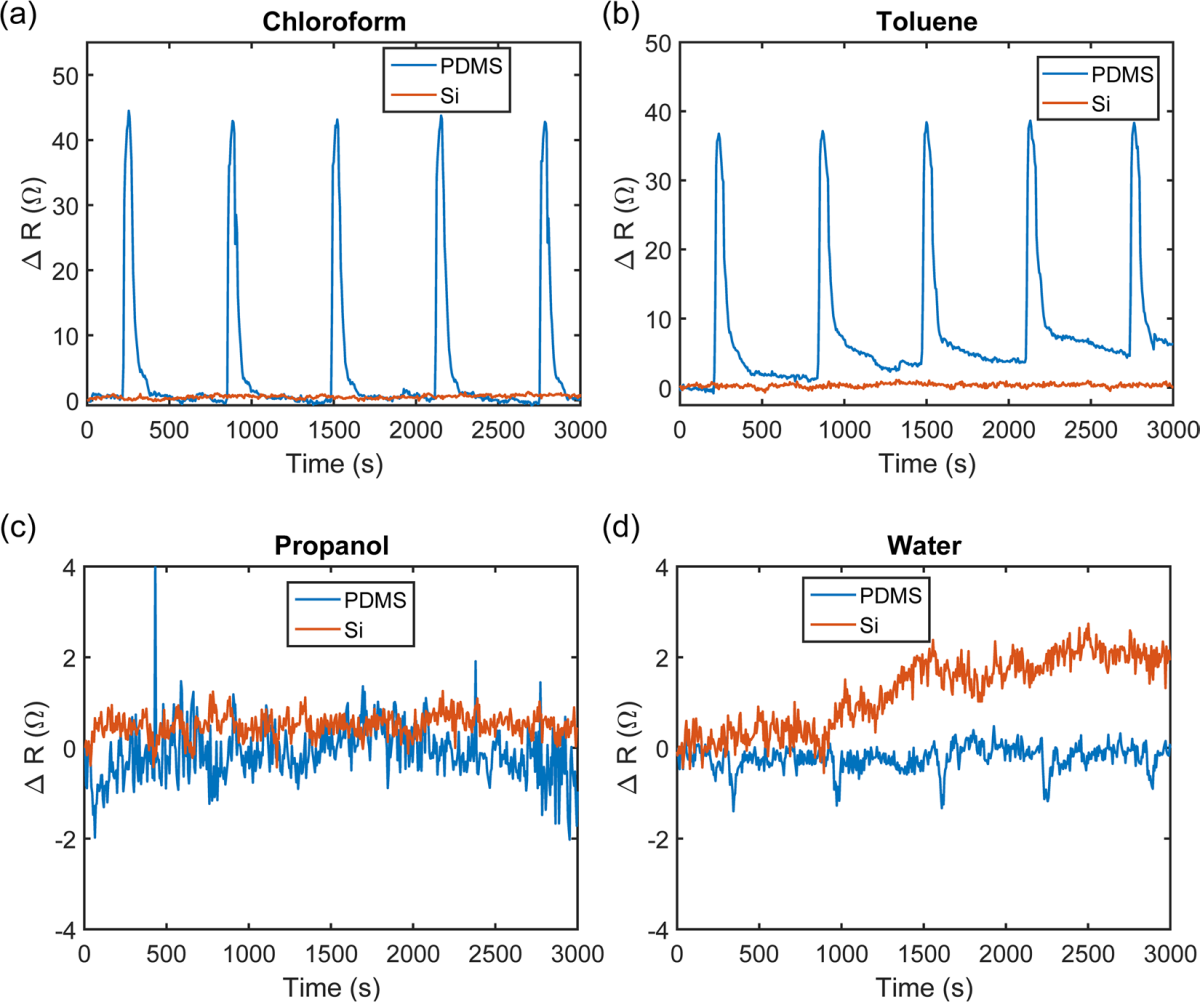
Change in resistance with time normalized to the initial resistance at *t* = 0 of the resistor fabricated on PDMS (blue line) in comparison with silicon (red line) toward 4 different vapors (**a**) Chloroform shows clear response and recovert (**b**) Toluene also causes the nano-resistor to become less conductive but the recovery is poor (**c**) IPA shows negligible change in resistance (**d**) Water shows small decrease in resistance in the nano-resistor on PDMS at the vapor injection cycles.

When subjected to chloroform vapor (4.40 × 10^5^ ppm), the resistance of the devices fabricated on PDMS increased by ~43 Ω, whereas no change was detected on the Si nano-resistor, as illustrated in Fig. 4(a). As chloroform is a halogenated organic solvent with a high solubility parameter of 9.2 cal^½^ cm^−3/2^ towards PDMS, it can cause swelling in the PDMS and impart mechanical stress onto the embedded metal nanostructures^44^. Such swelling induced stress would not be generated onto the nano-sensors deposited on Si substrate, resulting in unchanged resistance value. We also observed the full recovery of the devices as the value of *R* dropped back to the baseline within 3 minutes after dry air was flushed through the system. Likewise, we found that PDMS swells in the presence of saturated toluene vapor (3.80 × 10^4^ ppm), recording a Δ*R* of ~37 Ω, as shown in Fig. 4(b). However, not only were the devices unable to make a full recovery within 10 minutes, but the baseline *R* also increased after each toluene pulse, as shown in the same Fig. 4(b). This could be attributed by the high affinity of toluene towards the silcones and the higher boiling point (b.p.) of toluene (~110 °C) compared to chloroform (b.p. 61.5 °C), which renders the removal and evaporation of the toluene molecules more difficult once they diffuse through the PDMS. To achieve recovery of the sensor resistance, the device can be heated after toluene exposure to facilitate the out-diffusion of the toluene molecules from the PDMS. In the case of IPA (Fig. 4(c)), we did not see any change within the 30 seconds pulse intervals. This could be due to either the insignificant stretching of the resistor or the slower swelling response of the PDMS as IPA is more polar in nature, leading to a stronger repulsion of IPA molecules by the hydrophobic PDMS surface. We observed from Fig. 4(d) that the value of *R* of the devices decreased slightly with water vapor, where a Δ*R* of ~−1.5 Ω was measured. This can be explained by the fact that the nano-resistors embedded in the polymeric network were already subjected to stress due to prior swelling responses and this could have created discontinuities in the film. As water vapor may get adsorbed onto the Au or Ti (with a strong gettering effect) grains, the discontinuities will be filled and hence the conductivity of this VOC sensor could be improved^45^. At each injection point of water vapor in the chamber, the water molecules shorted the mesh on the PDMS resulting in a more conductive pathway between the contacts. In contrast, this was not observed with the more rigid Si reference device, which instead demonstrated a gradual drift in *R* by +2 Ω over the measurement time. Nevertheless, these findings suggest that ambient humidity does not have a strong impact on the sensing response. Our results also showed that PDMS has a preferential selectivity towards compounds of high hydrophobicity and solubility parameter values close to PDMS.

**Figure 5:**
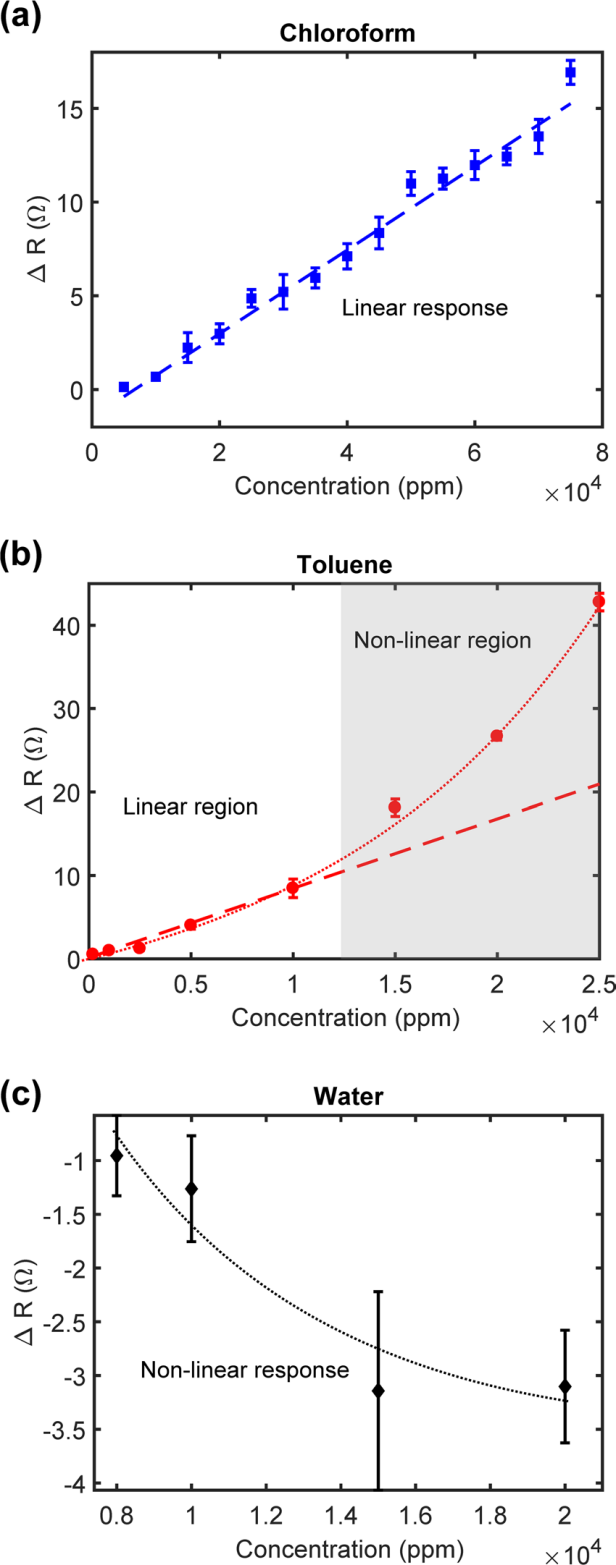
Electrical responses of the device at different concentrations of (**a**) Chloroform shows a linear increasing response over the concentration range investigated (**b**) Toluene initially causes linear rise in the resistance, but at higher concentrations, the response is no longer linear (**c**) Water shows a small decrease in resistance and has negligible change after 1.4 × 10^4^ ppm. Five measurements were recorded for each device, the mean of the measurements is shown as the center point and the standard deviation is represented by the error bars.

We then characterized the nano-resistors fabricated on PDMS against chloroform, toluene and water vapor at various concentrations as shown in Fig. 5. We diluted the saturated vapor of the corresponding solvent from the bubbler with dry air at varying flow rates (See Supporting Information Tables S1, S2 and S3 for dilution data). Chloroform vapor of 7.5 × 10^4^ ppm was injected into the system until the value of *R* reached a steady state. The measurements were repeated with lower concentrations of the same vapor at 5000 ppm intervals. We detected down to 1 × 10^4^ ppm of chloroform with an applied voltage of 1 mV. Over the concentration range tested, the nano-resistors exhibited a linear response toward chloroform with a sensitivity of 2.18 × 10^−4^ Ω/ppm as shown in Fig. 5(a). The VOC sensors shown in this work responded to toluene vapor concentrations as low as 1000 ppm. Additionally, concentrations as low as 200 ppm of toluene vapor were detected when we improved the signal to noise ratio of the nano-resistor by increasing the applied voltage by a further 0.5 mV to 1.5 mV. We observed a linear response to toluene vapor at concentrations up to 10000 ppm followed by a non-linear increase in Δ*R* as shown in Fig. 5(b). Despite a prior study reporting that PDMS swells more in chloroform than toluene liquid, we observed the converse when performing the measurements in these two organic vapors^46^. The more sensitive behavior of the nano-resistor on PDMS towards toluene vapor could be correlated to the closer resemblance of Hildebrand value: δ = c^1/2^ = −(*U*/*V*)^1/2^, where c (cal/cm^3^) represents the cohesive energy value, *U* represents the molar internal energy (cal/mol) and *V* represents the molar volume (cm^3^/mol)^15,47^. Another contributing mechanism could be the non-polarity of toluene with electronegativity value of 3.86 eV compared to of 5.51 eV for chloroform. This lower electronegativity of toluene may result in strong affinity of toluene vapor, which displayed a stronger interaction with PDMS in our experiments^46^. Water vapor only affects the device’s electrical responses above 8000 ppm threshold value. However, no clear trend was observed over the concentration range we investigated.

To ascertain that this VOC sensor can be used for real-time monitoring with negligible effects from environmental changes, we calibrated the effects of temperature on Δ*R*. Our experiments showed that the value of *R* of the nano-resistors increased by only ~2 Ω for every 10 °C increase in the surrounding temperature (See Supporting Information Figure S2). This change in resistance is negligible compared to the Δ*R* values observed for chloroform and toluene. If required, the effects of temperature drift could be taken into account by pairing the gas sensor device with a temperature sensor fabricated on silicon substrates. Further optimization is still required to improve the overall performance of the device as a gas sensor. The sensitivity of the gas sensor could be enhanced by the design of the resistor, material used for the resistor and possibly the shape of the polymeric layer. The selectivity of the gas sensor could be achieved by functionalization of the PDMS layer^48^. We also propose to apply this setup (nano-resistors fabricated directly onto the active layer) to other sensing materials for the discrimination of gas species present at trace levels. It is noted that, as for all resistive-type gas sensors, known techniques to provide cross-sensitivity analysis against interferent species in a gas mixture could be applied to the proposed approach, such as those based on multisensor arrays and pattern recognition (often referred to as electronic nose)^49,50^. From these experiments however, we have demonstrated a nanofabrication strategy to pattern conductive metallic structures of dimensions as small as 400 nm. Our method could be modified for applications that require a flat interface or standalone structures below 50 nm on elastomers such as plasmonic waveguides on polymers, which could be also accomplished by self-assembly of metal nanoparticles^51–53^. Other applications for high resolution features on polymers include tunable grating structures, and stamps with embedded metal for nanoimprinting. To achieve higher resolution features, we reduced the surface roughness of the sacrificial layer by sputtering the Al layer directly on freshly cleaved mica instead of Si substrates for template stripping (See Supporting Information Figure S3). Consequently, the rms roughness of the sacrificial Al layer reduced by ~80 folds to as low as ~0.1 nm. To confirm the viability of our method for high resolution patterning on PDMS, we subsequently patterned Au/Ti features of ~25 nm in width on the flatter Al surface and successfully transferred them onto PDMS (See Supporting Information Figures S4 and S5 for AFM images).

## Conclusion

In this work, we have shown that conductive and high resolution metallic structures can be fabricated onto elastomeric materials such as PDMS. Our approach in adopting a metallic sacrificial layer in transferring patterns defined by lithographic techniques, which overcomes the shortcomings of previously reported fabrication strategies. As the samples were subjected to minimal stress, devices made by this method remained conductive at the end of the process. We have applied the proposed method to demonstrate a nano-resistor with mesh design with features as small as ~400 nm embedded in PDMS for VOCs detection as a proof of concept. Using PDMS as both a substrate and an active layer simultaneously, our sensors showed preferential selectivity towards toluene and chloroform but did not swell in response to IPA and water within the exposure time. This study could also contribute to the stretchable electronics community in the design of high performance devices through miniaturization of existing works on elastomers, potentially paving the way for new applications in the future. We anticipate this proof of concept sensing mechanism could be further developed into technologies for continuous monitoring of a range of both vapors and liquids, and into fabrication of devices for point of care (PoC) diagnostics.

## Methods

### Sacrificial layer deposition and patterning

Al sacrificial layers (~150 nm thick) were deposited on Si wafers in a DC magnetron sputtering system (Metallifier sputter coater, Precision Atomics, Cambridge, UK) using a 100 mm diameter and 3 mm thick Al target (purity 99.95%). The deposition conditions for Al were 3.5 × 10^−3^ mbar sputtering pressure, 30 sccm of 99.999% purity Ar and 100 W power, giving a deposition rate of ~6 nm/min. The Si wafers were then diced into 1 cm length square chips, and PMMA (A4 950 K) was spin-coated on the chips at 5500 rpm and baked at 200 °C for 2 mins on a hotplate. The design of the sensor consisted of 2 pads of 1 × 1 mm and separated by 1 mm. The pads were connected by a mesh with a period 16 µm repeated by 62 times laterally and 20 times perpendicularly. The mesh pattern was produced using EBL (Nanobeam nB5 80 kV, Cambridge, UK) with a dose of 6 C/m^2^. After exposure the PMMA was developed in a 1:3 mixture of MIBK:IPA for 20 s, rinsed for 15 s in IPA and then dried with nitrogen. Au (50 nm) and Ti (4 nm) were subsequently evaporated in an e-beam evaporator (Model PVD75, Kurt J Lesker, Jefferson Hills, PA, USA) with the respective deposition rate of 0.04 nm/s and 0.03 nm/s. Excess Au/Ti were lifted-off in acetone with sonication, followed by cleaning with acetone, IPA and nitrogen drying. 95% (3-mercaptopropyl) trimethoxysilane purchased from Sigma-Aldrich (Dorset, UK) was used to prepare a solution of 60 mM of the silane in absolute ethanol. The chips were then immersed in the prepared solution for 2 hours and subsequently rinsed with clean absolute ethanol.

### Elastomer preparation and pattern transfer

PDMS (Sylgard 184) was purchased from Dow Corning (Seneffe, Belgium); the base and curing agent were mixed in the mass ratio 10:1 and degassed in a vacuum desiccator for 2 hours. The resulting polymer was then spin-coated at 1000 rpm for 40 s on the Au/Ti patterned chips to obtain a PDMS thickness of approximately 170 µm. PDMS curing was carried out in ambient cleanroom conditions for 72 hours. Standard HCl (37% by mass) was purchased from Sigma-Aldrich (Dorset, UK) and diluted with de-ionized (DI) water (volume ratio 1:6), which was then used to etch the sacrificial Al layer for 5 hours. The released PDMS with the embedded Au patterns was then washed in DI water and dried with nitrogen. The resulting PDMS was then briefly treated with oxygen plasma for 10 seconds.

### Device characterization and sensor performance

The devices fabricated on PDMS were sputter coated with a thin Au/Pd (palladium) layer for SEM imaging. SEM images of the devices were acquired using a LEO GEMINI 1530VP field-emission system (Zeiss, Oberkochen, Germany) with an acceleration voltage of 2 kV. Tapping mode AFM (Agilent 5500 scanning probe microscope, Palo Alto, CA, USA) was used to characterize the roughness inherited from the sacrificial Al surface. Solvents including chloroform, toluene and IPA were purchased from Fisher Scientific (Loughborough, UK) for gas sensing measurements.

## Electronic supplementary material

Supplementary material

## Data Availability

Data generated or analyzed during this study are included in this published article (and its Supplementary Information files).
