# Author Correction: Nanofabrication of Conductive Metallic Structures on Elastomeric Materials

**DOI:** 10.1038/s41598-018-30954-0

**Published:** 2018-09-11

**Authors:** Edward K. W. Tan, Girish Rughoobur, Juan Rubio-Lara, Nikhil Tiwale, Zhuocong Xiao, Colin A. B. Davidson, Christopher R. Lowe, Luigi G. Occhipinti

**Affiliations:** 1 Department of Engineering, University of Cambridge, Cambridge, CB3 0FA UK; 2 Microsystems Technology Laboratories, Massachusetts Institute of Technology, Cambridge, MA 02139 USA; 3 Nanoscience Centre, University of Cambridge, Cambridge, CB3 0FF UK; 4 Institute of Biotechnology, University of Cambridge, Cambridge, CB2 1QT UK

Correction to: *Scientific Reports* 10.1038/s41598-018-24901-2, published online 26 April 2018

In the Supplementary material file originally published with this Article, an additional image was included that obscured Table S1. This error has been corrected in the Supplementary material that now accompanies the Article.

