# Supplementary material for: Nanofabrication of Conductive Metallic Structures on Elastomeric Materials
Source: Sci Rep. 2018 Apr 26;8:6607. doi: 10.1038/s41598-018-24901-2 (PMC5920093; doi:10.1038/s41598-018-24901-2)
Supplement: Supplementary file 1 — Supplementary material [file 41598_2018_24901_MOESM1_ESM.docx]

Nanofabrication of Conductive Metallic Structures on Elastomeric Materials – *Supplementary material*

Edward Tan^1^, Girish Rughoobur^1,2^, Juan Rubio Lara^3^, Nikhil Tiwale^3^, Zhuocong Xiao^3^, Colin A.B. Davidson^4^, Christopher R. Lowe^4^ and Luigi G. Occhipinti^1^

1. Department of Engineering, University of Cambridge, Cambridge, CB3 0FA, UK.

2. Massachusetts Institute of Technology, Cambridge, MA 02139, USA.

3. Nanoscience Centre, University of Cambridge, CB3 0FF, UK.

4. Department of Chemical Engineering and Biotechnology, University of Cambridge, Cambridge, CB3 0AS, UK.


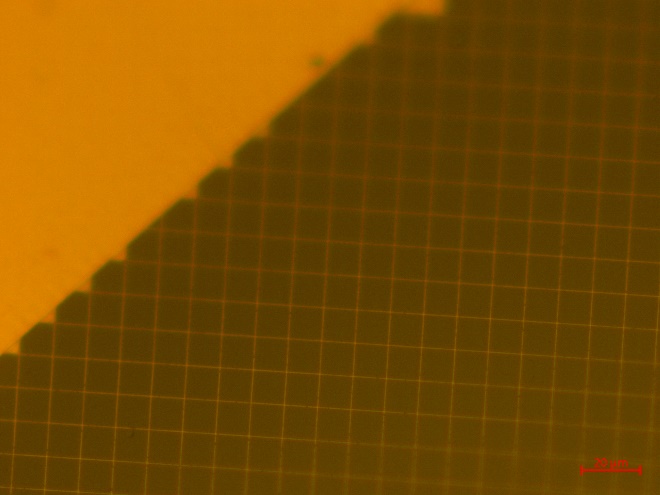


(a)

20 µm


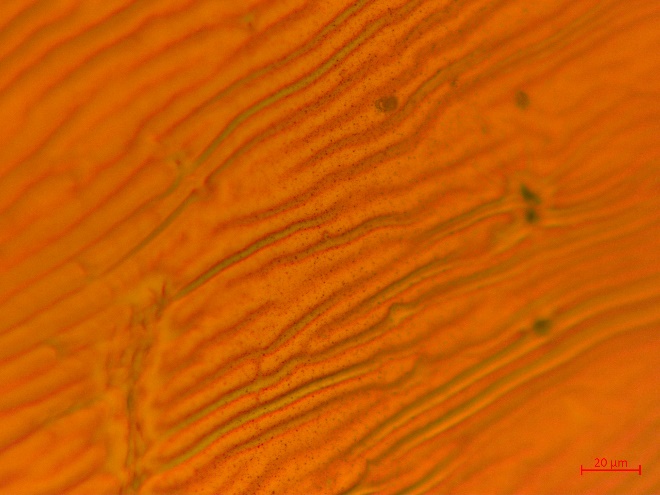


(b)

20 µm

Figure S1 – (a) Optical image of the gas sensor fabricated on PDMS after curing at room temperature (b) Optical image of gold film fabricated on PDMS after curing at 80 °C for 40 minutes.

Table S1 – Concentration of chloroform vapor at different air flowrate dilutions

| Concentration (ppm) | MFC1 (sccm) | MFC2 (sccm) | MFC3 (sccm) | MFC4 (sccm) |
| --- | --- | --- | --- | --- |
| 0 | 0 | 500.00 | 0 | 0 |
| 10000 | 0 | 485.71 | 0 | 14.29 |
| 20000 | 0 | 471.42 | 0 | 28.58 |
| 30000 | 0 | 457.13 | 0 | 42.87 |
| 40000 | 0 | 442.84 | 57.16 | 0 |
| 50000 | 0 | 428.55 | 71.45 | 0 |
| 60000 | 0 | 414.26 | 85.74 | 0 |
| 70000 | 0 | 399.97 | 100.03 | 0 |
| 80000 | 0 | 385.68 | 114.32 | 0 |
| 90000 | 0 | 371.39 | 128.61 | 0 |
| 100000 | 0 | 357.10 | 142.90 | 0 |
| 110000 | 0 | 342.81 | 157.19 | 0 |
| 120000 | 0 | 328.52 | 171.48 | 0 |
| 130000 | 0 | 314.23 | 185.77 | 0 |
| 140000 | 0 | 299.94 | 200.06 | 0 |
| 150000 | 0 | 285.65 | 214.35 | 0 |
| 160000 | 0 | 271.36 | 228.64 | 0 |
| 170000 | 0 | 257.07 | 242.93 | 0 |
| 180000 | 0 | 242.78 | 257.22 | 0 |
| 190000 | 0 | 228.49 | 271.51 | 0 |
| 200000 | 0 | 214.20 | 285.80 | 0 |
| 210000 | 0 | 199.91 | 300.09 | 0 |
| 220000 | 0 | 185.62 | 314.38 | 0 |
| 230000 | 0 | 171.33 | 328.67 | 0 |
| 240000 | 0 | 157.04 | 342.96 | 0 |
| 250000 | 0 | 142.75 | 357.25 | 0 |
| 260000 | 0 | 128.46 | 371.54 | 0 |
| 270000 | 0 | 114.17 | 385.83 | 0 |
| 280000 | 0 | 99.88 | 400.12 | 0 |
| 290000 | 0 | 85.59 | 414.41 | 0 |
| 300000 | 0 | 71.30 | 428.70 | 0 |
| 310000 | 0 | 57.01 | 442.99 | 0 |
| 320000 | 0 | 42.72 | 457.28 | 0 |
| 330000 | 0 | 28.43 | 471.57 | 0 |
| 340000 | 0 | 14.14 | 485.86 | 0 |
| 349900 | 0 | 0 | 500.00 | 0 |

Table S2 - Concentration of toluene vapor at different air flowrate dilutions

| Concentration (ppm) | MFC1 (sccm) | MFC2  (sccm) | MFC3  (sccm) | MFC4 (sccm) |
| --- | --- | --- | --- | --- |
| 0 | 0 | 500.00 | 0 | 0 |
| 1000 | 0 | 487.12 | 0 | 12.88 |
| 2000 | 0 | 474.24 | 0 | 25.76 |
| 3000 | 0 | 461.36 | 0 | 38.64 |
| 4000 | 0 | 448.48 | 0 | 51.52 |
| 5000 | 0 | 435.60 | 64.4 | 0 |
| 6000 | 0 | 422.72 | 77.28 | 0 |
| 7000 | 0 | 409.84 | 90.16 | 0 |
| 8000 | 0 | 396.96 | 103.04 | 0 |
| 9000 | 0 | 384.08 | 115.92 | 0 |
| 10000 | 0 | 371.20 | 128.80 | 0 |
| 11000 | 0 | 358.32 | 141.68 | 0 |
| 12000 | 0 | 345.44 | 154.56 | 0 |
| 13000 | 0 | 332.56 | 167.44 | 0 |
| 14000 | 0 | 319.68 | 180.32 | 0 |
| 15000 | 0 | 306.80 | 193.20 | 0 |
| 16000 | 0 | 293.92 | 206.08 | 0 |
| 17000 | 0 | 281.04 | 218.96 | 0 |
| 18000 | 0 | 268.16 | 231.84 | 0 |
| 19000 | 0 | 255.28 | 244.72 | 0 |
| 20000 | 0 | 242.40 | 257.60 | 0 |
| 21000 | 0 | 229.52 | 270.48 | 0 |
| 22000 | 0 | 216.64 | 283.36 | 0 |
| 23000 | 0 | 203.76 | 296.24 | 0 |
| 24000 | 0 | 190.88 | 309.12 | 0 |
| 25000 | 0 | 178.00 | 322.00 | 0 |
| 26000 | 0 | 165.12 | 334.88 | 0 |
| 27000 | 0 | 152.24 | 347.76 | 0 |
| 28000 | 0 | 139.36 | 360.64 | 0 |
| 29000 | 0 | 126.48 | 373.52 | 0 |
| 30000 | 0 | 113.60 | 386.40 | 0 |
| 31000 | 0 | 100.72 | 399.28 | 0 |
| 32000 | 0 | 87.84 | 412.16 | 0 |
| 33000 | 0 | 74.96 | 425.04 | 0 |
| 34000 | 0 | 62.08 | 437.92 | 0 |
| 35000 | 0 | 49.20 | 450.80 | 0 |
| 36000 | 0 | 36.32 | 463.68 | 0 |
| 37000 | 0 | 23.44 | 476.56 | 0 |
| 38000 | 0 | 10.56 | 489.44 | 0 |
| 38820 | 0 | 0 | 500.00 | 0 |

Table S3 - Concentration of water vapor at different air flowrate dilutions

| Concentration (ppm) | MFC1 (sccm) | MFC2  (sccm) | MFC3  (sccm) | MFC4 (sccm) |
| --- | --- | --- | --- | --- |
| 0 | 0 | 500.00 | 0 | 0 |
| 1000 | 0 | 479.39 | 0 | 20.61 |
| 2000 | 0 | 458.78 | 0 | 41.22 |
| 3000 | 0 | 438.17 | 61.83 | 0 |
| 4000 | 0 | 417.56 | 82.44 | 0 |
| 5000 | 0 | 396.95 | 103.05 | 0 |
| 6000 | 0 | 376.34 | 123.66 | 0 |
| 7000 | 0 | 355.73 | 144.27 | 0 |
| 8000 | 0 | 335.12 | 164.88 | 0 |
| 9000 | 0 | 314.51 | 185.49 | 0 |
| 10000 | 0 | 293.90 | 206.10 | 0 |
| 11000 | 0 | 273.29 | 226.71 | 0 |
| 12000 | 0 | 252.68 | 247.32 | 0 |
| 13000 | 0 | 232.07 | 267.93 | 0 |
| 14000 | 0 | 211.46 | 288.54 | 0 |
| 15000 | 0 | 190.85 | 309.15 | 0 |
| 16000 | 0 | 170.24 | 329.76 | 0 |
| 17000 | 0 | 149.63 | 350.37 | 0 |
| 18000 | 0 | 129.02 | 370.98 | 0 |
| 19000 | 0 | 108.41 | 391.59 | 0 |
| 20000 | 0 | 87.80 | 412.20 | 0 |
| 21000 | 0 | 67.19 | 432.81 | 0 |
| 22000 | 0 | 46.58 | 453.42 | 0 |
| 23000 | 0 | 25.97 | 474.03 | 0 |
| 24000 | 0 | 5.36 | 494.64 | 0 |
| 24260 | 0 | 0 | 500.00 | 0 |


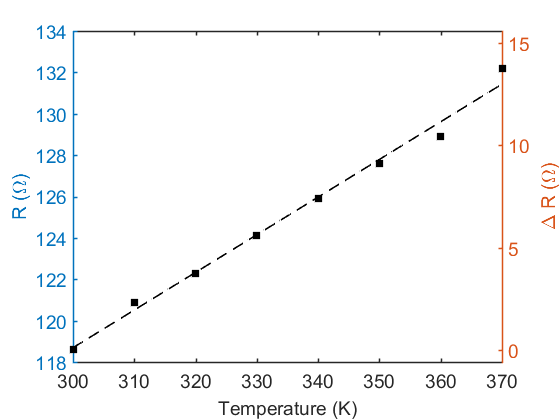


Figure S2 – Evolution of device resistance, *R*, (left axis) measured with temperature and the equivalent change in resistance, Δ*R*, (right axis) of the nano-resistor at different temperatures, showing a sensitivity of 0.18 Ω/K.


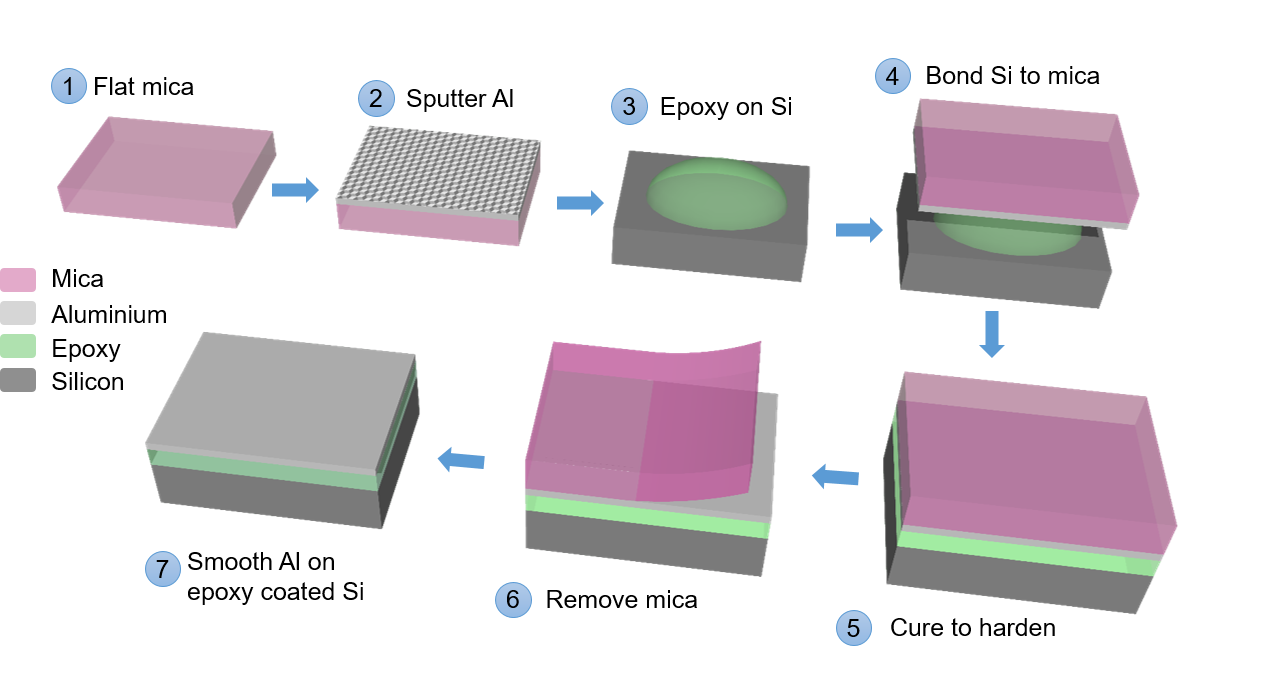


Figure S3 – Template stripping method using mica as substrate, Al as sacrificial layer and epoxy to bond Al on Si thereby achieving a flat surface onto which high resolution lithography can be patterned.


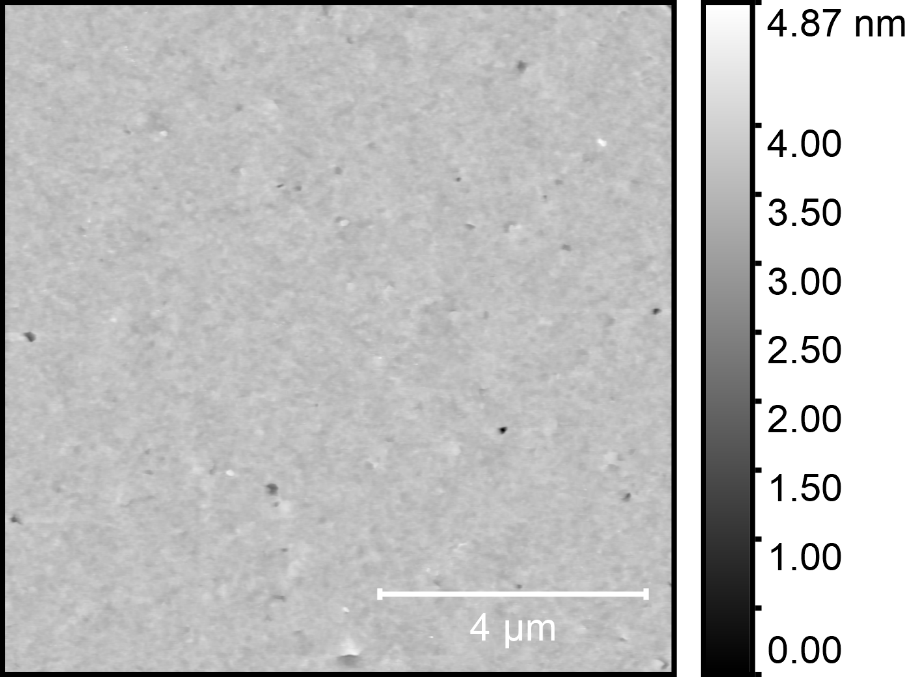


Figure S4 – AFM scan (amplitude) of Al sputtered on mica demonstrating a smooth surface with rms roughness of 0.114 nm.


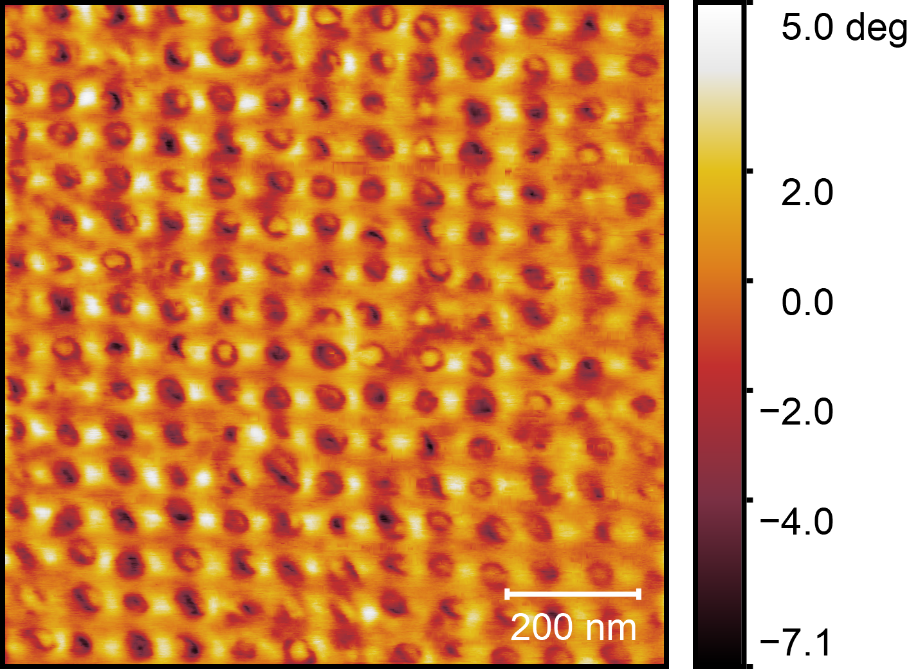


Figure S5 – AFM scan (phase) of the transferred high-resolution Au features (~40 nm) on PDMS fabricated using the template stripping method of flat Al on mica.
